# Supplementary material for: Drug repurposing to combat multidrug-resistant hookworm
Source: Biol Open. 2026 Feb 12;15(2):bio062380. doi: 10.1242/bio.062380 (PMC12937922; doi:10.1242/bio.062380)
Supplement: Supplementary information [file biolopen-15-062380-s1.pdf]

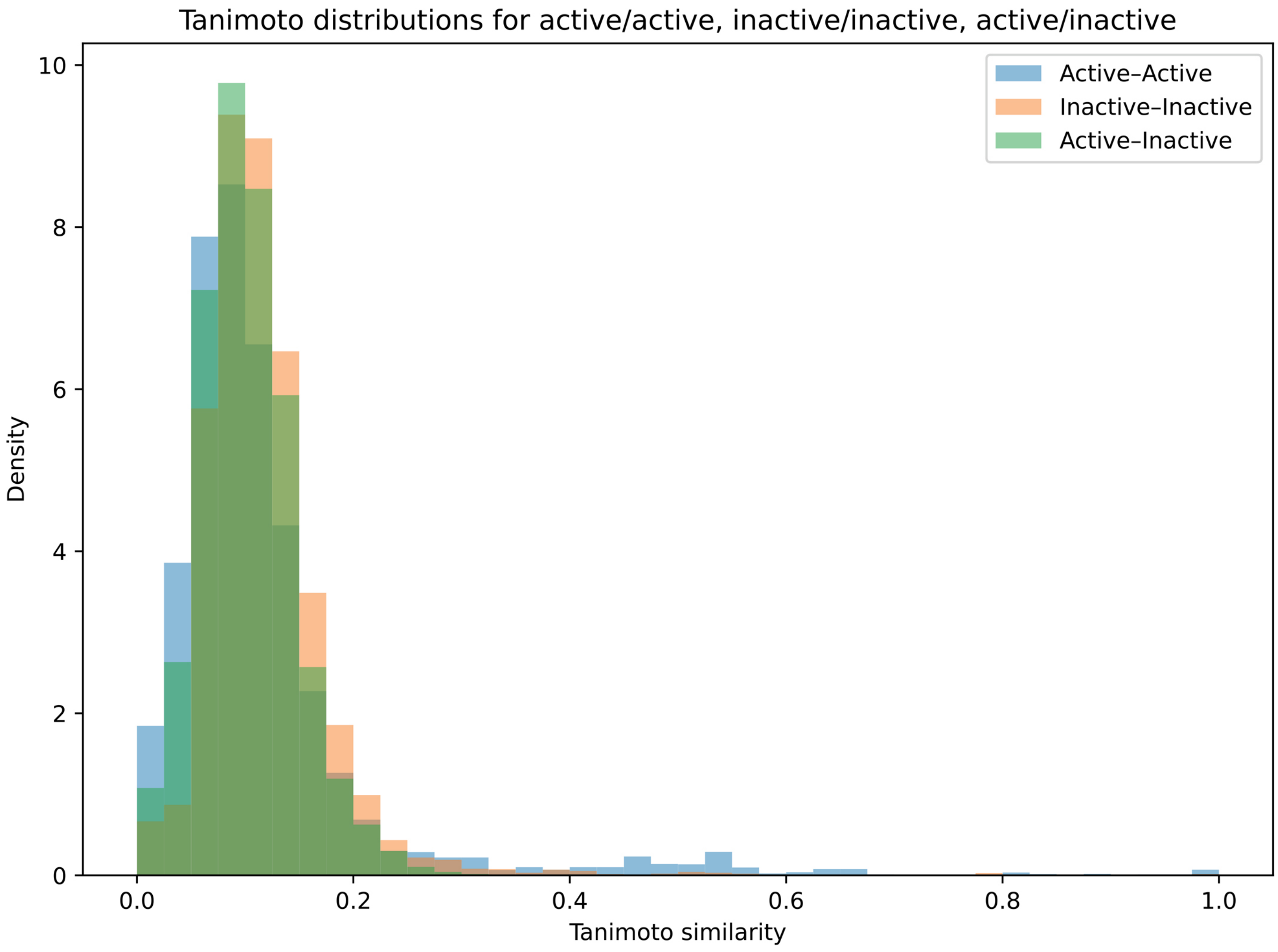

Fig. S1. Tanimoto similarity distributions of the active and inactive datasets. Distribution of pairwise Tanimoto similarity coefficients calculated from atom-pair fingerprints (ChemmineR sdf2ap). The histogram (with overlaid density curve) summarizes all pairwise similarities among compounds in the library and illustrates the overall spread of structural similarity within the dataset.

Biology Open • Supplementary information

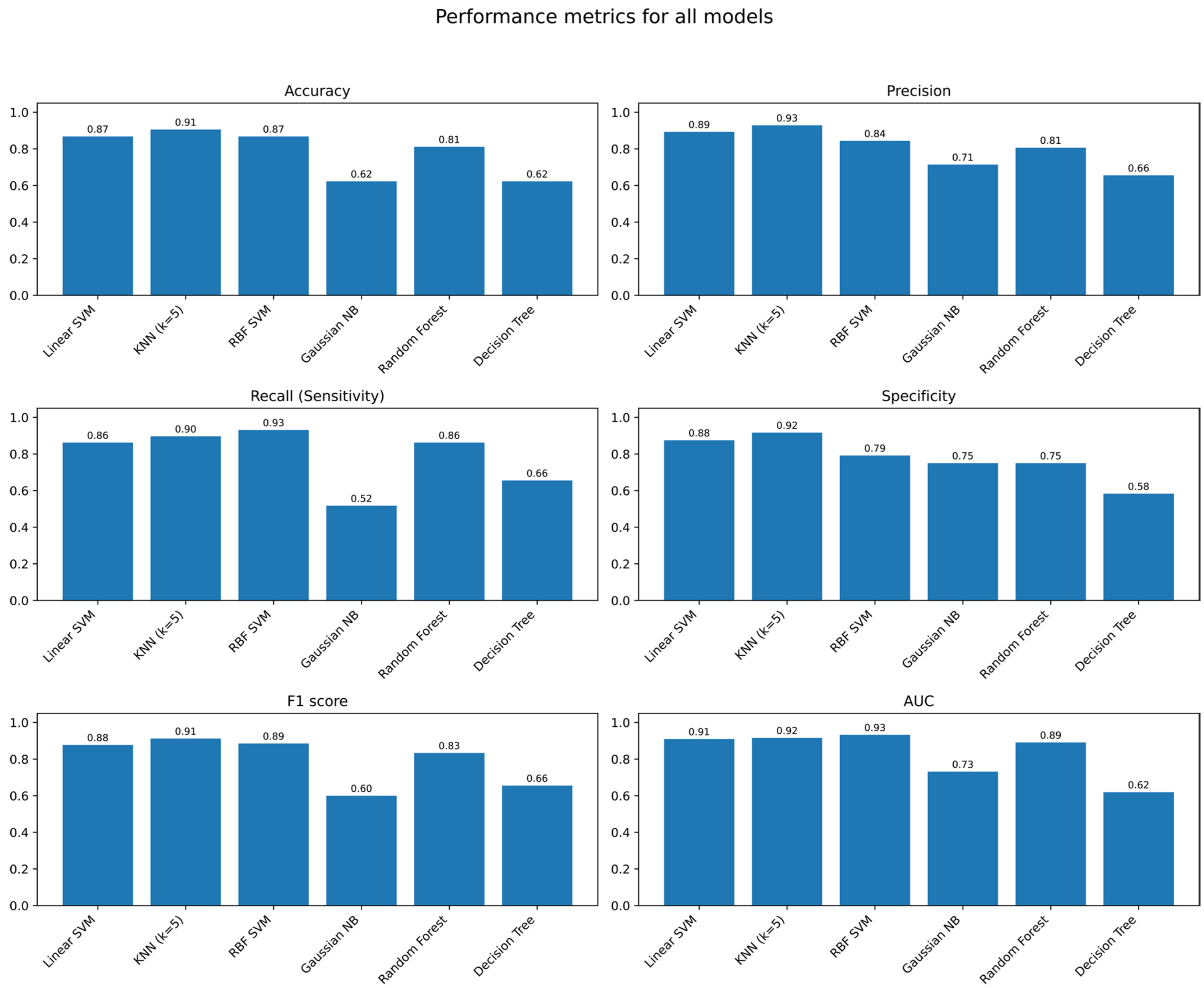

Fig. S2. Performance metrics for all classifiers evaluated in this study. Summary plots showing sensitivity (recall), specificity, precision, accuracy, F1 score, and area under the receiver operating characteristic curve (AUC) for each classifier used to distinguish active from inactive compounds. Each bar represents the average performance of a given classifier over the evaluation procedure described in the Methods.

Biology Open • Supplementary information

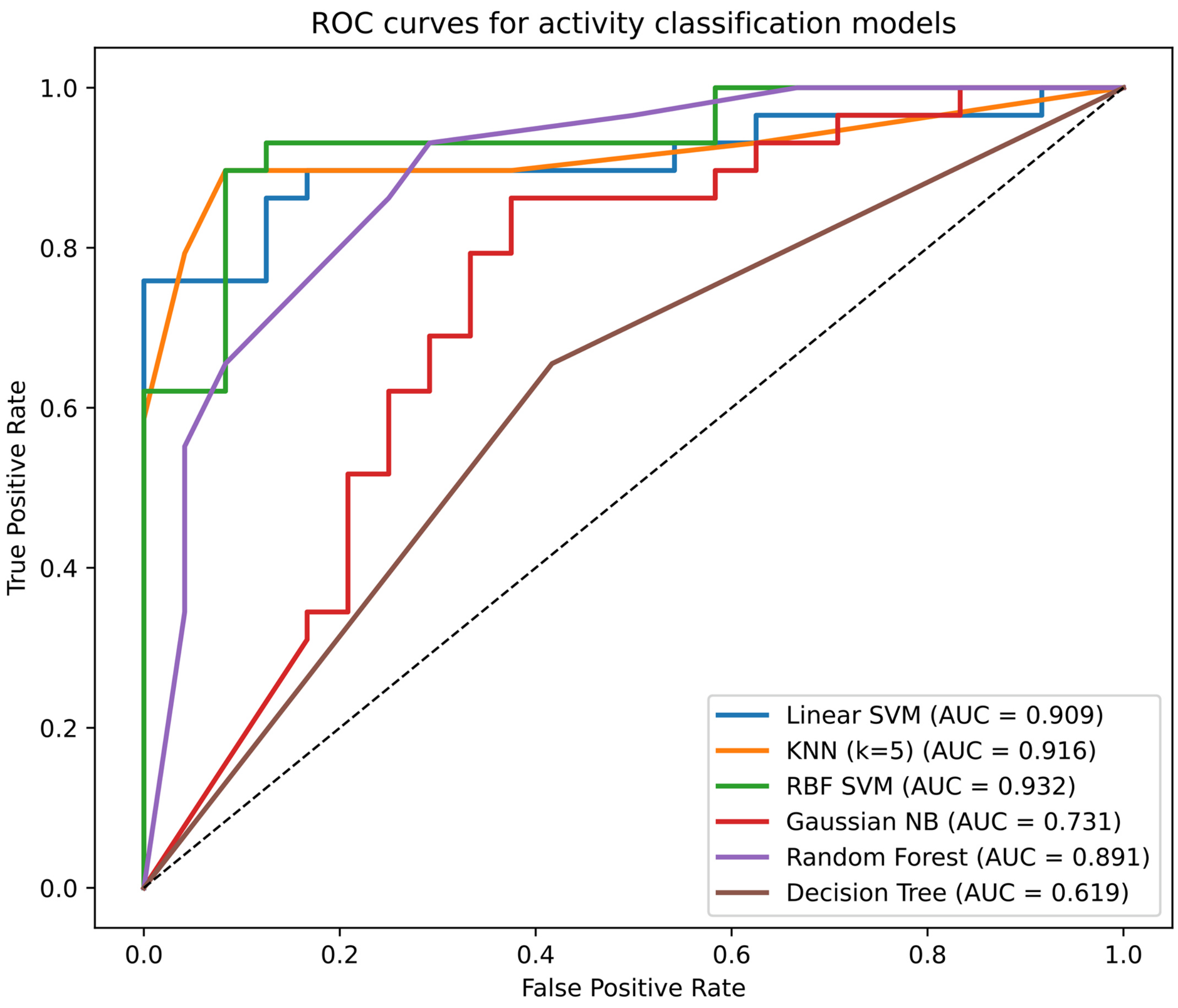

Fig. S3. Receiver operating characteristic (ROC) curves for all classifiers. ROC curves for each classifier evaluated on the active versus inactive classification task. True positive rate (sensitivity/recall) is plotted against false positive rate across a range of decision thresholds. Curves closer to the top-left corner indicate better discriminatory performance. The corresponding AUC values are reported for each classifier to provide a single summary measure of ROC performance.

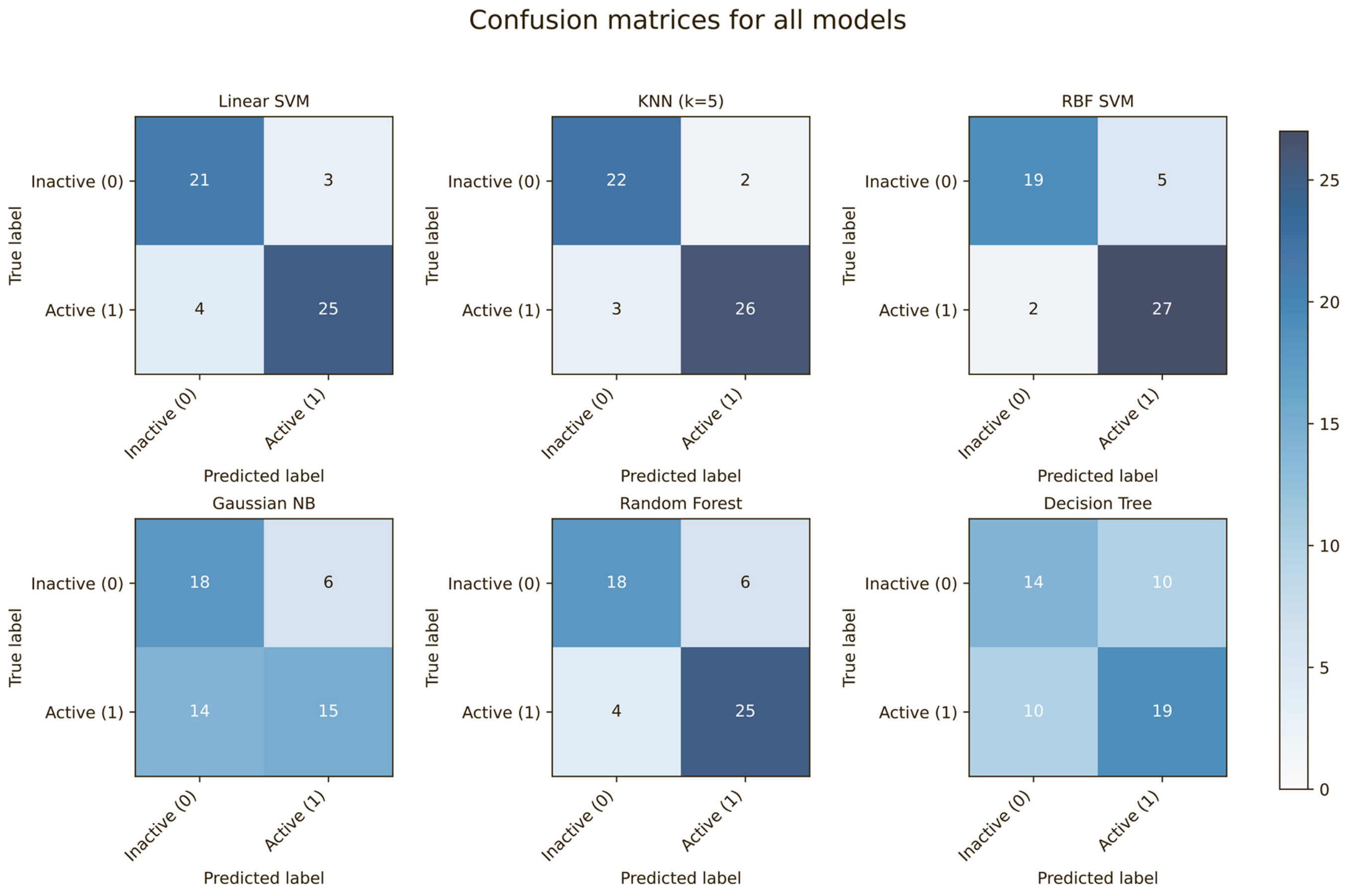

Fig. S4. Confusion matrices for each classifier. Multi-panel confusion matrix figure showing the number of true positives, false positives, true negatives, and false negatives for each classifier used to predict active versus inactive compounds. Each panel corresponds to a single classifier, with cell values indicating the observed counts and color intensity reflecting the magnitude of each cell. Together, these matrices provide a detailed view of the types of errors made by each model and complement the summary performance metrics shown in Figs S2-S3.

Biology Open • Supplementary information

Table S1. Complete list of all compounds included in the study, classified as active or inactive in the primary screen.

Available for download at
https://journals.biologists.com/bio/article-lookup/doi/10.1242/bio.062380#supplementary-data

Table S2. Complete list of all molecular descriptors calculated and used for the modeling of the active compound dataset.

Available for download at
https://journals.biologists.com/bio/article-lookup/doi/10.1242/bio.062380#supplementary-data

Table S3. Complete list of all molecular descriptors calculated and used for the modeling of the inactive compound dataset.

Available for download at
https://journals.biologists.com/bio/article-lookup/doi/10.1242/bio.062380#supplementary-data

Table S4. Predicted activities of all DrugBank-approved molecules. Complete list of all DrugBank-approved compounds evaluated by our models, showing the predicted activity from each individual classifier and the overall consensus (majority vote) classification for each compound.

Available for download at
https://journals.biologists.com/bio/article-lookup/doi/10.1242/bio.062380#supplementary-data

Biology Open • Supplementary information
